# 6G-Powered Efficient Resource Control through IRS-UE Association

**DOI:** 10.3390/s23218713

**Published:** 2023-10-25

**Authors:** Ali Alqahtani, Ashu Taneja, Jarallah Alqahtani, Nayef Alqahtani

**Affiliations:** 1 Department of Networks and Communications Engineering, College of Computer Science and Information Systems, Najran University, Najran 61441, Saudi Arabia; asalqahtany@nu.edu.sa; 2 Chitkara University Institute of Engineering and Technology, Chitkara University, Rajpura 140401, Punjab, India; 3 Department of Computer Science, College of Computer Science and Information Systems, Najran University, Najran 61441, Saudi Arabia; jaalqahtani@nu.edu.sa; 4 Department of Electrical Engineering, College of Engineering, King Faisal University, Al-Hofuf 31982, Al-Ahsa, Saudi Arabia; nmalqahtani@kfu.edu.sa

**Keywords:** 6G, IoT, IRS, IRS-UE association, sum rate

## Abstract

The widespread popularity of live streaming, cloud gaming, mobile video streaming, and many real-time applications relies on high-speed data to ensure low latency and seamless user experience. This large high-speed data demand has led to the development of next-generation or sixth-generation (6G) communication technology. It aims to offer high-speed communication support to multiple applications and interactive services simultaneously. But the vulnerability of node communication to the changing propagation environment often leads to call drops, data loss, and high latency. This paper presents a 6G-enabled wireless network that makes use of multiple intelligent reflecting surfaces (IRSs). The distributed IRSs enhance the robustness of transmission but the increased overhead owing to multiple IRSs is the main challenge. To overcome this, efficient resource control is introduced, which associates sets of IRSs to user equipment (UE). An algorithm, namely IUABP (IRS-UE association based on pilots), is proposed; it offers selective resource control. Furthermore, the performance of the distributed IRS system is evaluated based on the achievable sum rate for different IRS numbers, reflecting elements, and transmit powers. We observed that the proposed association scheme offers an improvement of 30% in the achieved sum rate using *N* = 50 and *R* = 5 at a transmit power of 12 dBm. We also discuss the comparison with two other association schemes, namely, distance-based association and random association.

## 1. Introduction

Global mobile traffic is increasing at an alarming rate. As per the estimation by ITU-R, it is estimated that by the end of 2030, global mobile traffic will reach 5016 exabytes per month [1]. This is attributed to mobile video applications, like YouTube and Netflix. as well as high-screen resolution on mobile devices. Mobile cloud services, machine-to machine (M2M) communications, rich-video applications are proliferating, making it hard for fifth-generation (5G) wireless technology to meet high data requirements [2,3]. The emergence of new electronic products, VR glasses, smart watches, and wearable electronics involves intelligent applications and interactive services placing huge data demands on communication systems [4,5]. This has led to the development and evolution of future wireless technology, moving beyond 5G or 6G. These next-generation mobile communication systems aim to support high-speed communications simultaneously on multiple devices [6]. High-frequency millimeter waves (mmWaves) and terahertz (THz) waves offer improved capacity but due to shorter wavelengths and high attenuation, they have limited beamforming capabilities, with the communicating nodes often blocked by physical objects [7,8]. Thus, the vulnerability of wireless communications to harsh wireless propagation environments, including dead zones and large obstacles, is a main challenge. It is very important to ensure wireless transmission is robust against harsh environments, blockages, and large obstacles to prevent signal losses, call drops, and transmission delays [9].

An intelligent reflecting surface (IRS) is a promising 6G technology that offers a smart radio environment and enhances wireless transmission robustness [10]. An IRS consists of an array of passive reflecting elements that smartly reflect the incident electromagnetic (em) waves in the direction of the intended user [11]. In other words, it is possible to increase the signal power of edge user nodes by manipulating the reflection coefficient of the IRS [12]. The phase shifts of reflecting elements can be smartly controlled by means of the IRS controller and new propagation paths can be created through channel redesigning [13]. These additional paths result in an improved signal-to-interference-plus-noise ratio (SINR). IRS technology can also mitigate high-frequency signal losses or attenuation in mmWave or THz bands [14]. The high mobility of user nodes causing the Doppler effect or low transmission rates can be taken care of with IRS technology. IRSs can also help to reduce multipath fading and shadowing effects through efficient reflection control, providing desired beamforming capabilities [15]. High transmission rates can be achieved and user nodes in dead zones can be served [16]. IRSs can be integrated with spatial modulation techniques to achieve enhanced spatial multiplexing, improved network coverage, reduced interference, and maximum spectrum utilization [17]. Support for multiple access via index modulation in IRS-aided systems can also yield better performance [18]. The convergence with other emerging technologies, like blockchain, the Internet of Things (IoT), and deep learning, has been helpful in obtaining desired characteristics in wireless sensor networks [19] and unmanned aerial vehicles [20].

Due to the potential of IRSs, IRS technology is being used for various applications. By enabling smart reflections, it can be used in intelligent transportation systems (ITSs) for real-time traffic management, thereby reducing traffic congestion and improving traffic control efficiency [21]. The data from the sensors can be redirected and analyzed for environmental monitoring [22]. IRSs also find applications in underwater communications in the Internet of Underwater Things for smart ocean transportation [23]. The limited battery lives of IoT-enabled consumer devices can be enhanced with IRS-enabled energy harvesting [24]. With the ability to control the propagation environment, IRSs can be employed for indoor localization and navigation systems [25]. In wireless communications, IRSs are used to enhance the reliability of wireless communications for various communication scenarios. IRS-aided communications in single-input-single-output (SISO) and multiple-input-single-output (MISO) transmission models were investigated in [26,27], respectively. Multiple-input-multiple-output (MIMO) communication scenarios have been explored in [28] for multiple users [29]. An IRS-aided massive MIMO system is highlighted in [30], where dynamic reflection is obtained through phase shift optimization. The achievable rate of the system is maximized using the channel information for optimized phase shifts. Apart from this, several IRSs are being used in the communication framework to assist user transmissions with the distributed IRS (D-IRS) communication system [31]. The centralized IRS deployment is compared with the distributed IRS system for performance evaluation in [32]. The sum rate of the edge users is evaluated and it is observed that the D-IRS system outperforms the counterpart. The role of double IRS in improving the spectral efficiency of the multi-user system is investigated in [33]. By optimizing the phase shift and precoding matrices, the performance of the system is evaluated. The energy efficiency of the IRS-aided system is analyzed in [34], in which the energy consumption is reduced using the on–off switching of the IRS. The performance of D-IRS is investigated in [35] for an indoor scenario involving a THz propagation environment. In [36], the authors use multiple IRSs in vehicular networks to achieve extended coverage and enhanced quality-of-service (QoS). The sum rate of the vehicular network is increased by optimizing the beamforming vectors using max–min SINR criteria.

But the main challenge in the D-IRS system is the significant increase in the number of propagation channel paths, owing to the use of multiple IRSs. This, in turn, will increase the channel estimation overhead and add to the system complexity [37]. Also, for a large number of reflecting surfaces, the need for accurate beamforming results in additional overhead. The literature includes papers that deal with channel estimation in IRS-aided systems. The on–off switching of different IRS elements in different time slots is considered in [38] for estimating the cascaded channel in the IRS system. In [39], the phase shifts of the IRS are obtained using the discrete Fourier transform (DFT) matrix to obtain the channel estimates. IRS-UE association is introduced to overcome the channel estimation and beamforming overheads. By associating an IRS with a node or a group of nodes, the remaining IRSs can be turned off. This also results in reduced energy overhead and improves the system’s performance. In random association, IRS is randomly selected for each user node and assigned to it for optimal performance. Distance-dependent association selects the nearby IRS and assigns it to the user equipment (UE) [40]. IRS selection addresses the channel estimation overhead and enables efficient resource control.

In this paper, a distributed IRS-aided communication system is considered, where multiple IRSs assist in the transmission of multiple user nodes. An algorithm, namely IUABP (IRS-user association based on pilots), is proposed, which associates a subset of IRSs for each user node. Through optimal pilot allocation, an IRS that increases the SINR at each user node is selected and assigned to that node. The performance of the system is evaluated for an achieved sum rate using different IRS numbers, reflecting elements, and transmit powers.

### Contributions and Outcomes

High-speed and low-latency demands of various machine-to-machine (M2M) communications involving real-time data have led to the evolution of communication technology, moving toward next-generation technology. The main challenge in 6G networks involves the effect of the propagation environment on wireless transmission. This paper presents a 6G-enabled wireless network, in which multiple IRSs assist the communication between the BS and user nodes. The novel contributions of this paper are as follows:

- A distributed IRS system model is considered to serve the user nodes in dead zones, under harsh environments, with the aid of multiple IRSs. The distributed IRS system is compared with the centralized IRS system with one centralized IRS in the system.
- The increased overhead challenge in a multi-IRS system is overcome by proposing a resource control algorithm, namely IUABP (IRS-UE association based on pilots). The IUABP algorithm assigns subsets of IRSs to user equipment (UE), based on efficient pilot allocation.
- After the selective association, the effective channel between the BS and user nodes via selected IRSs is estimated against all IRS channels.
- The mathematical formulations of the signal processing involved in estimating the channel and the evaluation of the system sum rate is provided.
- The performance of the system with the proposed IUABP algorithm is evaluated for the sum rate achieved under different transmit powers, different reflecting elements per IRS, and different IRS numbers.
- The proposed algorithm is also compared for performance with a distance-based association scheme and a random association scheme.

The mathematical notations and variables used in this paper are tabulated in Table 1 below.

## 2. System Model

**Figure 1:**
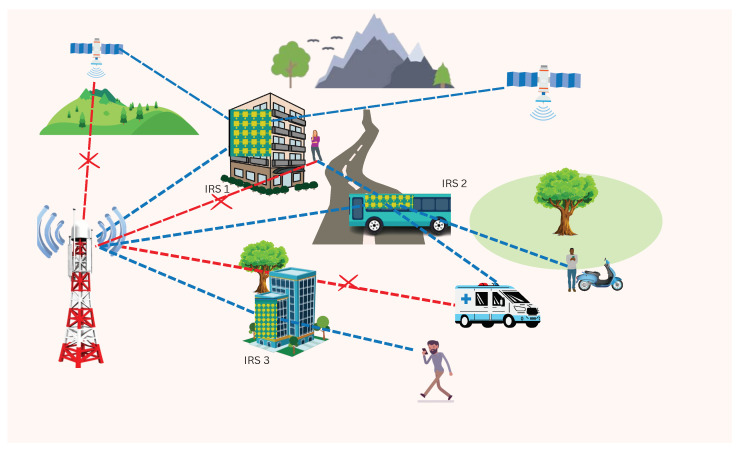
Illustration of a system model with a BS serving a number of users through distributed IRSs.

Consider a communication model in which a multi-antenna BS serves *K* number of single antenna user nodes. The BS has a uniform linear array with size *M*. The communication between the BS and the user nodes is assisted by *R* IRSs distributed in the communication network as shown in Figure 1. Let R denote the set of *R* IRSs, such that $R=\left\{1,2,\ldots ,R\right\}$, which are installed under the coverage of BS. Each IRS has *N* reflecting elements arranged as uniform linear arrays with *N* = $N_{1}$×$N_{2}$. All IRSs are connected to the same IRS controller. The incoming signals on the IRS are reflected independently by each IRS element with a unique phase shift. Let us define a matrix $\Theta_{r}=diag\left(A_{r}\theta_{r}\right)$, denoting the reflection coefficients of the *r*th IRS. Let $A_{r}$ be the common amplitude coefficient of the *r*th IRS and $\theta_{r}$ be the phase shift vector. Let $\left\{\theta_{1}^{r},\theta_{2}^{r},\ldots ,\theta_{N}^{r}\right\}$ be the phase shifts induced by the *N* reflecting elements of *r*th IRS, such that $\theta_{r}={\left[\theta_{1}^{r},\theta_{2}^{r},\ldots ,\theta_{N}^{r}\right]}^{T}$. $A_{r}$ can take two sets of values, $A_{r}$ = 0 implies that the IRS is off while $A_{r}$ = 1 means that the IRS is on. $\theta_{n}^{r}=e^{j\phi_{n,r}}$ with $\phi_{n,r}\in \left(-\pi ,\pi \right)$ being the phase shift.

### 2.1. Channel Modeling

Let us suppose that the direct channel between BS and the user node *k* is $G_{k}\in C^{\left(M\times 1\right)}$ and the channel between the BS and the *r*th IRS is $Q_{r}\in C^{\left(M\times N\right)}$. The channel between the *r*th IRS and the *k*th user node is $h_{rk}\in C^{\left(N\times 1\right)}$. $Q={\left[Q_{1}^{T},Q_{2}^{T},\ldots ,Q_{R}^{T}\right]}^{T}\in C^{\left(RN\times M\right)}$ represents the channel from the BS to all the *R* IRSs. $h_{k}=\left[h_{1k}^{T},h_{2k}^{T},\ldots ,h_{Rk}^{T}\right]\in C^{\left(RN\times 1\right)}$ represents the channel between all the IRSs and the *k*th user node. The channel between IRSs and user nodes follows the Rician fading model, which is the sum of the line-of-sight (LoS) component and non-line-of-sight (NLoS) component given below [41]

(1) $$h_{rk}=\sqrt{\frac{\xi_{rk}\vartheta_{rk}}{1+\vartheta_{rk}}}h_{rk}^{LoS}+\sqrt{\frac{\xi_{rk}}{1+\vartheta_{rk}}}h_{rk}^{NLoS}$$

where $\xi_{rk}$ is the Rician factor of the IRS-user link, $\vartheta_{rk}$ is the large-scale fading. $h_{rk}^{LoS}$ is the LoS component given by

(2) $$h_{rk}^{LoS}=\chi_{N}\left(\varphi_{rk},\varsigma_{rk}\right)$$

The array response vector at the *r*th IRS, consisting of *N* elements, arranged as a uniform linear array of $N_{1}$ × $N_{2}$, equally spaced apart by the *d* distance with the carrier wavelength λ, is given by  

$$\chi_{N}\left(\varphi ,\varsigma \right)=\left[1,e^{-j2\pi \frac{d}{\lambda }sin\varphi },\ldots ,e^{-j2\pi \left(N_{1}-1\right)\frac{d}{\lambda }sin\varphi }\right]$$

(3) $$⊗\left[1,e^{-j2\pi \frac{d}{\lambda }sin\varsigma },\ldots ,e^{-j2\pi \left(N2-1\right)\frac{d}{\lambda }sin\varsigma }\right]$$

The effective angles of departure (AODs)φ,ς are given by

(4) $$\varphi_{rk}=2\pi \frac{d}{\lambda }cos\left(\zeta_{rk}\right)sin\left(\mu_{rk}\right)$$

(5) $$\varsigma_{rk}=2\pi \frac{d}{\lambda }sin\left(\zeta_{rk}\right)sin\left(\mu_{rk}\right)$$

let $\zeta_{rk}$ and $\mu_{rk}$ be the azimuth and elevation AODs from the *r*th IRS to the *k*th user node. Similarly, the channels between the BS and the user nodes $G_{k}$ as well as the channels between BS and IRSs $Q_{k}$ follow the Rician distribution, as given below.

(6) $$G_{k}=\sqrt{\frac{\xi_{Bk}\vartheta_{Bk}}{1+\vartheta_{Bk}}}G_{k}^{LoS}+\sqrt{\frac{\xi_{Bk}}{1+\vartheta_{Bk}}}G_{k}^{NLoS}$$

(7) $$Q_{r}=\sqrt{\frac{\xi_{Br}\vartheta_{Br}}{1+\vartheta_{Br}}}Q_{r}^{LoS}+\sqrt{\frac{\xi_{Br}}{1+\vartheta_{Br}}}Q_{r}^{NLoS}$$

where $\vartheta_{Bk}$, $\vartheta_{Br}$ are the large-scale fading coefficients of the respective channel links, and $\xi_{Bk}$ and $\xi_{Br}$ are the Rician factors.

The block fading model is assumed, in which the coherence block of the $\tau_{c}$ length involves the $\tau_{p}$ interval for pilot transmission and the $\tau_{c}-\tau_{p}$ interval for data transmission. The channel remains constant and frequency=flat in each coherence block. The time division duplex (TDD) mode of operation is considered for the system model. Table 2 gives the comparison of Rician fading model over other models.

### 2.2. Pilot Transmission and Channel Estimation

In this phase, each node transmits the pilot signals to the BS. Let us suppose a set of $\tau_{p}$ training signals are designed, such that $\psi =\left\{\psi_{1},\psi_{2},\ldots ,\psi_{\tau_{p}}\right\}$ and $\|\psi_{t}{\|}^{2}=\tau_{p}$ for $t=1,2,\ldots ,\tau_{p}$.  

(8) $$\psi_{j}^{H}\psi_{k}=\left\{\begin{array}{c} \tau_{p}\;\;\;\;\;j=k\\ 0\;\;\;\;\;j\neq k\;\;\;\;\;\;\;\; \end{array}\right\}$$

The received pilot signal at the BS from the user node *k* is given by

(9) $$y_{B}^{p}=\sqrt{\alpha_{k}}\left(G_{k}+\sum_{r=1}^{R}Q_{r}^{H}\Theta_{r}h_{rk}\right)\psi_{k}+Z$$

where $\alpha_{k}$ is the pilot transmit power and *Z* is the receiver noise. For the BS to estimate the user node *k* channels, the received pilot signal is projected on $\psi_{k}^{H}$ as

(10) $$y_{kB}^{p}=\psi_{k}^{H}y_{B}^{p}$$

Since $\Theta_{r}=diag\left(A_{r}\phi_{r}\right)$ Equation (7) can be written as

(11) $$y_{B}^{p}=\sqrt{\alpha_{k}}\left(G_{k}+\sum_{r=1}^{R}A_{r}\phi_{r}Q_{r}^{H}diag\left(h_{rk}\right)\right)\psi_{k}+Z$$

where $H_{krB}=Q_{r}^{H}diag\left(h_{rk}\right)$ is the cascaded channel from the *k*th node to the BS. Also, $H_{kB}=\left(G_{k}+\sum_{r=1}^{R}Q_{r}^{H}\Theta_{r}h_{rk}\right)$ is the effective channel from the *k*th node to the BS.

The effective channel is estimated using the linear MMSE channel estimation method [46]

(12) $${\hat{H}}_{kB}=\left(E\left\{y_{kB}^{p*}H_{kB}\right\}y_{kB}^{p}\right)/E\left\{{\left|y_{kB}^{p}\right|}^{2}\right\}=q_{kB}y_{kB}^{p}$$

(13) $$q_{kB}=\left(E\left\{y_{kB}^{p*}H_{kB}\right\}\right)/E\left\{{\left|y_{kB}^{p}\right|}^{2}\right\}$$

$q_{kB}$ is also written as   

(14) $$q_{kB}=\frac{\sqrt{\alpha_{k}\tau_{p}}\gamma_{kB}}{\alpha_{k}\tau_{p}\sum_{j\in P_{k}}\gamma_{jB}+1}$$

where $P_{k}$ is the set of nodes assigned with the same pilot as node *k*.

(15) $$\gamma_{kB}=E\left\{{\left|H_{kB}\right|}^{2}\right\}$$

**Proof.**  Let us formulate the second and fourth moments of the effective channel as

(16) $$E\left\{{\left|H_{kB}\right|}^{2}\right\}=\eta_{kB}$$

(17) $$E\left\{{\left|H_{kB}\right|}^{4}\right\}=2\eta_{kB}-2tr\left(\Phi_{kB}^{2}\right)$$

where $\Phi_{kB}^{2}=\Theta_{r}^{H}\Theta_{r}$ and $\eta_{kB}=\vartheta_{kB}+tr\left(\Phi_{kB}\right)$.

The effective channels are mutually independent and mutually uncorrelated for $k\neq k^{{}^{\prime }}$.

(18) $$E\left\{H_{kB}H_{k^{\prime }B}\right\}=0$$

(19) $$E\left\{H_{kB}H_{k^{\prime }B}^{*}\right\}=0$$

Also, these follow the constraints

(20) $$E\left\{{\left|H_{kB}H_{k^{\prime }B}\right|}^{2}\right\}=\eta_{kB}\eta_{k^{\prime }B},k\neq k^{\prime }$$

The estimated channel has zero mean and variance $\delta_{kB}$, given as

(21) $$\delta_{kB}=E\left\{{\left|{\hat{H}}_{kB}\right|}^{2}\right\}$$

(22) $$\delta_{kB}=\sqrt{\alpha_{k}\tau_{p}}\gamma_{kB}q_{kB}$$

The channel estimation error is given by

(23) $$e_{kB}=H_{kB}-{\hat{H}}_{kB}$$

$e_{kB}$ has zero mean and variance $E\left\{{\left|e_{kB}\right|}^{2}\right\}=\eta_{kB}-\delta_{kB}$.

There is no correlation between the channel estimate and the channel estimation error.    □

### 2.3. Data Transmission

The data transmission phase involves transmitting information vector *s* to the *K* IoT nodes. Let us define $W=[w_{1},w_{2},\ldots ,w_{K}]$ as the precoding matrix, which follows the power constraint $\sum_{k=1}^{K}\left|\right|w_{k}{\left|\right|}^{2}\leq P_{t}$, with $P_{t}$ being the transmit power. Here, the downlink communication scenario is considered, in which the BS transmits the signals to the user nodes. Let $s={\left[s_{1},s_{2},\ldots ,s_{K}\right]}^{T}$ be the transmitted symbol, which is pre-coded with a precoding matrix or beamforming matrix $W=[w_{1},w_{2},\ldots ,w_{K}]$, such that $\sum_{k=1}^{K}\left|\right|w_{k}{\left|\right|}^{2}\leq P_{t}$, with $P_{t}$ being the transmit power [47]. The signal received at the *k*th node is given by

(24) $$y_{k}=\left(G_{k}^{H}+\sum_{r=1}^{R}h_{rk}^{H}\Theta_{r}Q_{r}\right)\sum_{k\in \eta_{k}}^{K}w_{k}s_{k}+z_{k}$$

where $\eta_{k}$ is the set of user nodes simultaneously served by the BS, such that $\eta_{k}\subset \left\{1,2,\ldots ,K\right\}$. This can be rewritten as

(25) $$y_{k}=\left(G_{k}^{H}+\sum_{r=1}^{R}h_{rk}^{H}diag\left(A_{r}\phi_{r}\right)Q_{r}\right)\sum_{k\in \eta_{k}}^{K}w_{k}s_{k}+z_{k}$$

(26) $$y_{k}=\left(G_{k}^{H}+\sum_{r=1}^{R}A_{r}\phi_{r}diag\left(h_{rk}^{H}\right)Q_{r}\right)\sum_{k\in \eta_{k}}^{K}w_{k}s_{k}+z_{k}$$

where $H_{Brk}=diag\left(h_{rk}^{H}\right)Q_{r}$ is the cascaded channel from BS to the *k*th node. Also, ($G_{k}^{H}+\sum_{r=1}^{R}A_{r}\phi_{r}H_{Brk})$ is the effective channel from BS to the *k*th node.

The signal-to-interference-plus-noise ratio at the *k*th node is given by

(27) $$\Upsilon_{k}=\frac{{∥\left(g_{k}^{H}+\sum_{r=1}^{R}h_{rk}^{H}\Theta_{r}Q_{r}\right)w_{k}∥}^{2}}{\sum_{j\in \eta_{k}}^{K}{∥\left(g_{k}^{H}+\sum_{r=1}^{R}h_{rk}^{H}\Theta_{r}Q_{r}\right)w_{k}∥}^{2}+\sigma_{k}^{2}}$$

The achievable rate $R_{k}$ is given by

(28) $$R_{k}=log_{2}\left(1+\Upsilon_{k}\right)$$

## 3. IRS-UE Association

**Figure 2:**
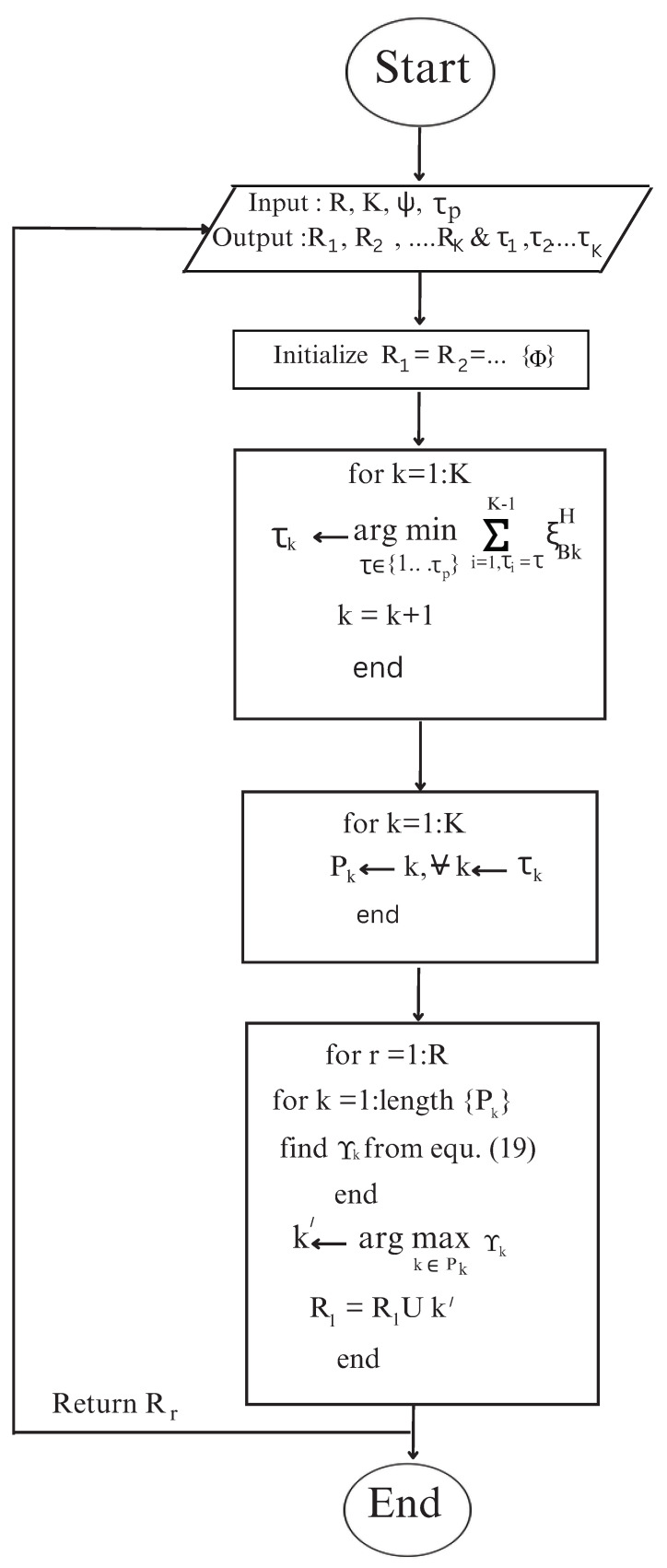
Flowchart of the proposed IUABP algorithm.

In the communication model presented in Section 2, multiple IRSs assist the communication between the BS and the user nodes. This results in high data rates, as the user signals blocked by obstacles in the dead zones are offered alternate paths via the use of several IRSs. However, this requires additional overhead owing to the increase in the number of propagation channels, which need accurate estimation for practical system applications. To overcome this additional overhead, this section presents an efficient mechanism that associates an IRS or set of IRSs to each user node. The IRS corresponding to a particular node will function while the rest of the IRSs will be switched off. This IRS-UE association will reduce the energy overhead due to additional IRSs being in an ’off’ state. Against using all IRSs at once for each user, the selected IRSs assist the user transmission. This will also reduce the number of channel paths to be estimated, resulting in improved system performance. The next subsection provides an algorithm, namely IUABP (IRS-UE association based on pilots). Also, the flowchart of the algorithm is presented in Figure 2.

### 3.1. IUABP Algorithm

**Figure 3:**
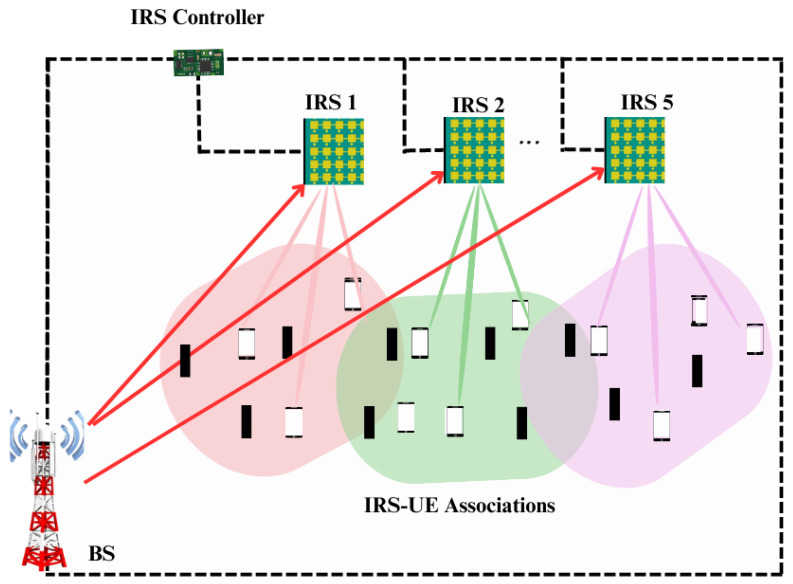
Simulation setup of the system model.

The considered communication framework deploys *R* IRSs at different locations in the geographical area. Let R denote the set of *R* IRSs, such that $R=\left\{1,2,\ldots ,R\right\}$, which are installed under the coverage of BS. The number of user nodes to be served is *K*. This algorithm associates an IRS or a set of IRSs to a particular node *k* based on minimum pilot interference and maximum SINR criteria. Each IRS can serve the number of user nodes limited by the pilot length. To serve a particular node *k*, only the serving IRS will be functional against all the IRSs in general. The steps of the algorithm are given in Algorithm 1. In the first phase, user nodes are assigned with the pilots from the set of mutually orthogonal pilot sets ψ of length $\tau_{p}$, $\psi =\left\{\psi_{1},\psi_{2},\ldots ,\psi_{t_{p}}\right\}$. For this, for each node *k*, a pilot $\tau_{k}$ is chosen in such a way that its transmission with the pilot transmit power $\alpha_{k}$ offers minimal interference at the BS. For the pilot allocation phase, it is assumed that all the IRSs are off and all the transmissions take place via the direct paths with large-scale fading $\xi_{Bk}$ between the BS and the *k*th node. This is repeated until pilots are allocated to all the nodes. After that, the nodes assigned to the same pilot $\tau_{k}$ are grouped together in a set $P_{k}$. After the formation of user sets with the same pilots, the next step associates a subset of IRSs to each node. For each user node in the set $P_{k}$, the received SINR $\Upsilon_{k}$ at node *k* from the BS transmission is calculated using Equation (19). The user node $k^{\prime }$ in $P_{k}$ with the maximum received SINR via the IRS *r* is found. The IRS *r* is assigned to the subset of IRSs $R_{k^{\prime }}$, which assists user $k^{\prime }$. This is repeated until all the IRS subsets for all the nodes are formed. Figure 3 illustrates the flow of operations of the algorithm. The key principle feature of this association scheme is that it results in reduced energy consumption, owing to a limited number of functional IRSs against all the IRSs. Since only a few IRSs are active at any given time to serve a specific node, the channel estimation overhead is reduced with fewer channel paths to estimate.
**Algorithm 1** IUABP algorithm.**Input** *R*, *K*, $\tau_{p}$, ψ**Output** $R_{1}$, $R_{2}$, …, $R_{K}$ and $\tau_{1}$, $\tau_{2}$, …, $\tau_{K}$    1. Initialization    $R_{1}=R_{2}=\ldots R_{K}=\left\{\Phi \right\}$    2. Pilot Assignment    for k=1:K    $\tau_{k}\leftarrow {arg\min}_{\tau \in \left\{1,\ldots ,\tau_{p}\right\}}\sum_{i=1,\tau_{i}=\tau }^{K-1}\xi_{Bk}^{H}$    k=k+1    end    3. Formation of user sets with the same pilots    for k=1:K    $P_{k}\leftarrow k$, $\forall k\leftarrow \tau_{k}$    end    4. IRS-UE Association    for r=1:R    for $k=1:length\left\{P_{k}\right\}$    find $\Upsilon_{k}$ from Equation (19)    end    $k^{\prime }\leftarrow {arg\;max\;}_{k\in P_{k}}\Upsilon_{k}$    $R_{k^{\prime }}=R_{k^{\prime }}\cup \left\{r\right\}$    end    **return** $R_{k^{\prime }}$


### 3.2. Implementation Complexity or Number of Estimated Links

The algorithm proposed in the previous subsection associates an IRS *r* with a particular user node *k*. Let us define a reflection matrix *F*, such that $f_{r,k}$ represents the r,kth element of the matrix $F\in {\left\{0,1\right\}}^{R\times K}$. The association scheme is such that an IRS *r* will serve node *k* if $f_{r,k}=1$. If ∀k, $f_{r,k}=0$, the IRS *r* will not serve node *k*, where $A_{r}=0$ and IRS will be in the off state. The number of estimated paths is given by

(29) $$L_{links}=\sum_{r=1}^{R}\sum_{k=1}^{K}f_{r,k}$$

The number of estimated paths is dependent on the number of ON IRSs as more than one IRS can support one node.

## 4. Results and Discussion

The system model presented in Section 2 is simulated in MATLAB and the results are presented in this section. Each simulation setup is run for $10^{4}$ realizations and 25 setups are taken. The simulation setup is illustrated in Figure 3. The BS is located at the origin (0, 0, 0) of a 100 × 100 m${}^{2}$ square area, where IRSs are randomly installed to meet the uniform user distribution in that region. The height of IRS is 5 m and the user nodes are of an average height of 1.5 m. The carrier frequency $f_{c}$ is 20 GHz. The simulation is carried out using the parameters tabulated in Table 3. The distributed IRS system has *R* number of IRSs in the considered geographical region, while there is only one central IRS in the coverage area in the case of the centralized IRS system. For the validation of results, the works carried out in [32,41] are taken as the benchmark for the distributed IRS system and centralized IRS system.

**Figure 4:**
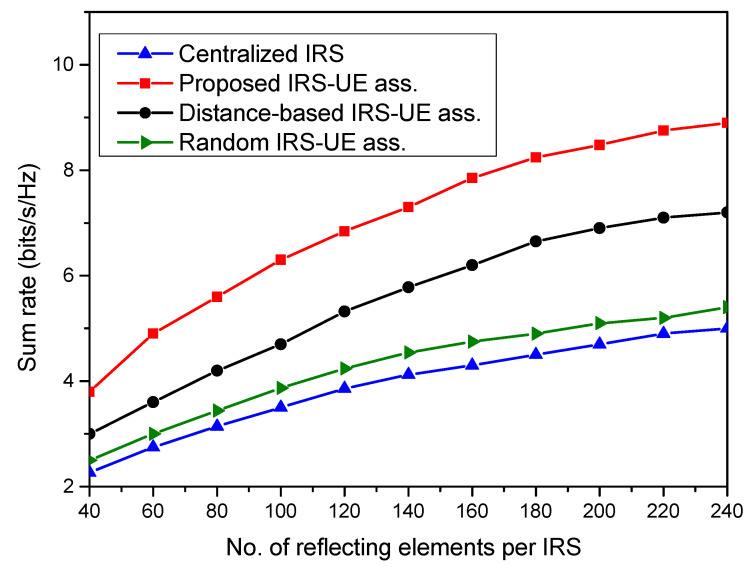
Sum rate performance of the centralized IRS system with the distributed IRS system for different IRS-UE associations.

Figure 4 depicts the sum rate performance of the distributed IRS system with IRS-UE association. Three different association schemes are considered to evaluate the system’s performance. The first is the proposed IUABP scheme, which associates sets of IRSs for serving particular user nodes, as defined in Section 3. The second is the distance-based IRS-UE association, which assigns the nearest IRS to each user node. And the third association scheme is a random association, in which any IRS is randomly used to serve any user. The sum rate achieved by the user nodes in the system with the association schemes is plotted and compared with the centralized system with no specific association. It is seen that the distributed IRS system with the proposed IUABP association scheme outperforms the other two association schemes. The gain in the sum rate achieved with random association in the distributed IRS system is 9% over the centralized IRS case, with *N* = 40. The proposed scheme achieves the highest sum rate of 6.3 bits/s/Hz in the system with *R* = 5 and *N* = 100 over 4.7 bits/s/Hz, with a distance-based scheme with the same system setup.

**Figure 5:**
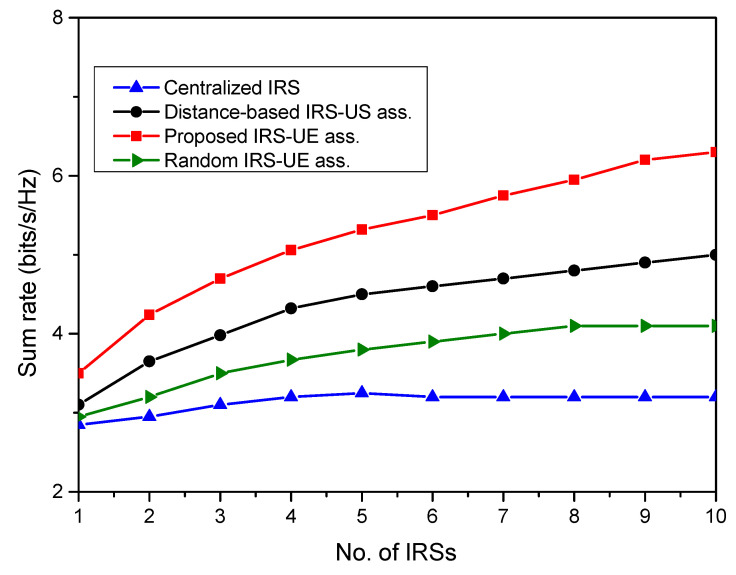
The sum rate as a function of different IRS numbers in multiple IRS systems.

The impact of varying the number of IRSs in the system on the system’s performance is carried out in Figure 5, keeping *N* fixed at 50 and $P_{t}$ at 12 dBm. In the simulation model, the IRSs vary from 1 to 10. It is visible from the figure that, as the number of IRSs increases, the sum rate achieved by the system improves. This is attributed to the increased user coverage with extra IRSs. With more IRSs, the IUABP scheme yields a higher sum rate. It is found to achieve a sum rate of 5.89 bits/s/Hz with *R* = 5 over 4.5 bits/s/Hz, with a distance-based scheme, and 3.8 bits/s/Hz with a random association. The performance of the centralized IRS system remains constant with change in *R* at 3.2 bits/s/Hz.

**Figure 6:**
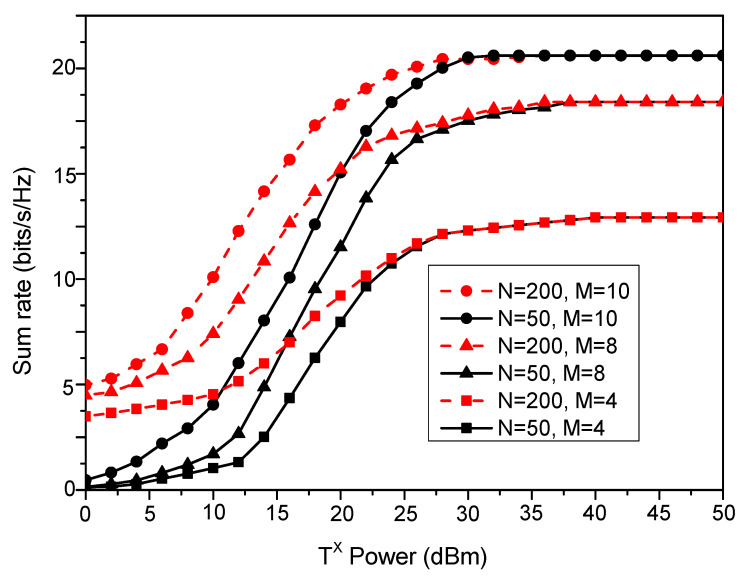
Sum rate performance of the distributed IRS system varies with changes in *N* and *M*.

Figure 6 demonstrates the sum rate of the system with varying transmit power values. The effect of varying BS antenna elements, *M*, and IRS reflecting elements, *N*, on the system sum rate with changes in transmit power is also depicted. It is observed that the maximum performance is achieved with IRSs with more reflecting elements and a BS with more antenna elements. For a fixed *M*, the variation in *N* yields an increasing sum rate for small to medium transmit power values, followed by a constant sum rate for large transmit power values. Also, for a fixed *N*, the increase in *M* yields a higher sum rate across all transmit power ranges. This is due to high spatial multiplexing and beamforming gains offered by increasing the antenna array at the BS.

**Figure 7:**
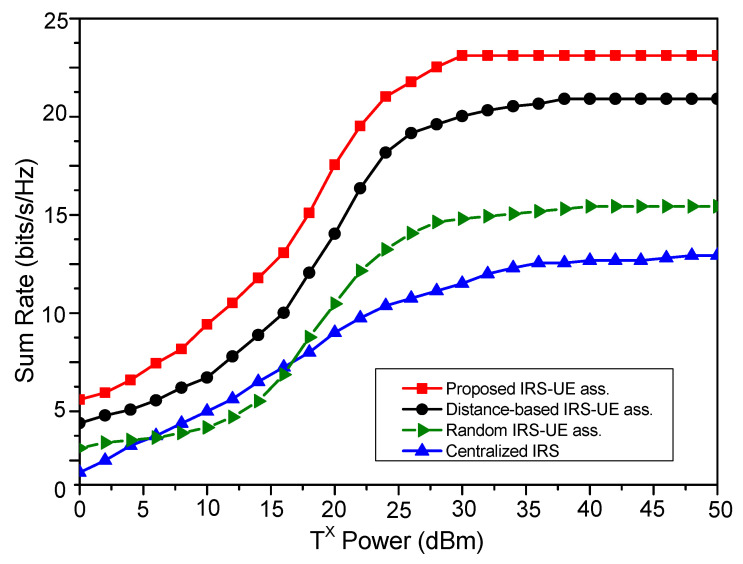
Comparison of the system sum rate for different IRS-UE association schemes.

**Figure 8:**
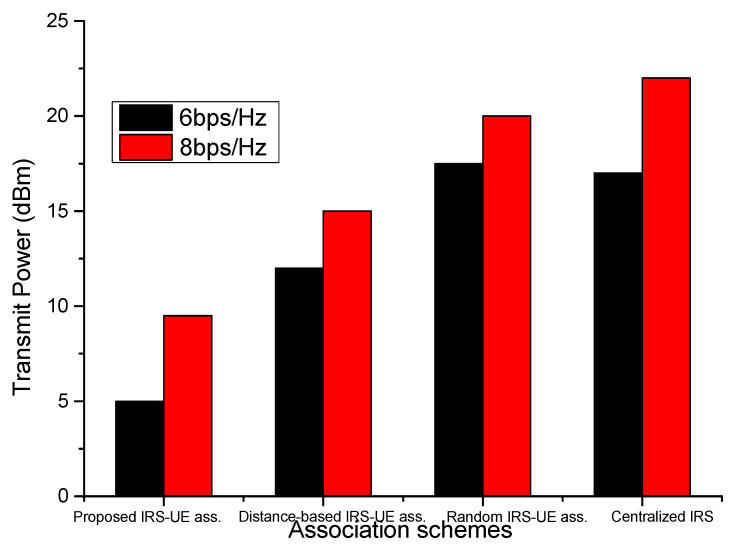
Power required to achieve target rates for different IRS-UE association schemes.

Figure 7 shows the performances of association schemes with transmit power changes. In the case of the centralized IRS system, the sum rate increases with an increase in transmit power. On the other hand, in the case of the distributed IRS, the sum rate first increases and then becomes constant. The proposed IUABP scheme improves the sum rate by a minimum of 10% over the distance-based association scheme for the entire transmit power range. However, the performance of the random association scheme is comparable to the centralized case for small transmit power values. The transmit power required to achieve a particular target rate for different association schemes is depicted in Figure 8. The transmit power required to achieve the target rate of 6 bits/s/Hz with a random association scheme stands at 17.5 dBm over 12 dBm in the distance-based scheme and 5 dBm for the proposed IUABP scheme. The transmit powers required for higher achievable rates are higher, but here, the proposed scheme outshines other schemes.

## 5. Conclusions

This paper presents a 6G-enabled distributed IRS system that offers network robustness by serving the dead zone user nodes. However, the challenge with multiple IRSs involves the additional overhead owing to channel estimation due to the large number of reflecting elements per IRS. To overcome this, this paper proposes an IUABP algorithm that associates subsets of IRSs to each UE based on pilots. The performance of the distributed IRS system is evaluated for the proposed algorithm and compared with two other association schemes, namely distance-based association and random association schemes. The gain in the sum rate achieved with random association in the distributed IRS system is 9% over the centralized IRS case, with *N* = 40. The proposed scheme achieves the highest sum rate of 6.3 bits/s/Hz in the system with *R* = 5, and *N* = 100 over 4.7 bits/s/Hz with the distance-based scheme. The impact of varying the number of IRSs in the system suggests that, as the number of IRSs increases, the sum rate achieved by the system improves. It is further observed that with the increase in transmit power, the sum rate increases in the case of the centralized IRS system. In the distributed IRS case, the sum rate first increases and then becomes constant. The proposed IUABP scheme improves the sum rate by a minimum of 10% over the distance-based association scheme for the entire transmit power range. For a fixed *M*, variations in *N* yield an increasing sum rate for small to medium transmit power values, followed by a constant sum rate for large transmit power values. Also, for a fixed *N*, the increase in *M* yields a higher sum rate across all transmit power ranges.

## Figures and Tables

**Table 1:** List of mathematical variables and notations.

Notation	Description
*R*	Number of IRSs
*N*	Number of reflecting elements per IRS
*M*	Number of antennas at the BS
*K*	Number of user equipment
$\Theta_{r}$	Reflection matrix of *r*th IRS
$G_{k}$	Direct channel between BS and the *k*th user node
$Q_{r}$	Channel between BS and the *r*th IRS
$h_{rk}$	Channel between the *r*th IRS and the *k*th user node
$H_{krB}$	Cascaded channel between the *k*th user node and the BS
$\alpha_{k}$	pilot transmit power of user *k*
$\vartheta_{Bk}$, $\vartheta_{Br}$	large-scale fading coefficients
$\xi_{Bk}$, $\xi_{Br}$	Rician factors
$\mu_{rk}$	elevation angles
$\zeta_{rk}$	azimuth angles
$\varphi_{rk}$, $\varsigma_{rk}$	Effective angle of departures
$\tau_{p}$	Pilot length
ψ	A set of $\tau_{p}$ pilot sequences
$\tau_{c}$	Length of coherence block
$P_{k}$	Set of nodes assigned with the same pilot
*Z*	Receiver noise
$P_{t}$	Total BS transmit power
$H_{kB}$	Effective channel from the *k*th node and the BS
${\hat{H}}_{kB}$	Estimates of the effective channel between BS and the *k*th node
$\Upsilon_{k}$	SINR at the *k*th node
$R_{k}$	Achievable rate
$y_{k}$	Received data signal at the *k*th node
$y_{B}^{p}$	Received pilot signal at the BS
$H_{BS}$	Height f BS from the ground plane
$H_{IRS}$	Height of IRS from the ground plane
$H_{UE}$	Height of the user equipment from the ground plane

**Table 2:** Comparison  of the considered Rician fading model against other potential models.

Ref.	Channel Fading Model Used	Advantage	Limitation
[42]	Uncorrelated Rayleigh fading	Commonly used and less complex	The spatial correlations among the reflecting elements of the IRSs are not considered.
[43]	Spatially correlated Rayleigh fading	More practical and realistic model. Considers the correlations among the IRS reflecting elements due to their geometric layouts, sizes, and inter-distances.	Computational complexity is more due to the requirement of covariance matrices.
[44]	Nakagami-m fading channel	Useful in real-world fading environments with varying multipath propagation degrees.	Channel characteristics are spatially homogeneous
[45]	Rician fading model	It provides a more realistic description of channel behavior when there is a strong LOS signal. Better SNR estimation	Not applicable in scenarios without LoS paths, estimation of the k-factor is challenging.

**Table 3:** Simulation parameters.

Parameters	Value	Parameters	Value
*N*	100	$\tau_{p}$	5
*R*	5	$P_{t}$	15 dBm
*M*	8	$H_{BS}$	10 m
$H_{IRS}$	5 m	$H_{UE}$	1.5 m
*K*	40	$\alpha_{k}$	10 mW
$f_{c}$	20 GHz	$\sigma^{2}$	−94 dBm
$\xi_{rk}$	5	$\xi_{Bk}$	5
$\xi_{Br}$	5		

## Data Availability

Not applicable.

## Abbreviations

The following abbreviations are used in this manuscript:

6G	sixth-generation
IRSs	intelligent reflecting surfaces
UE	user equipment
5G	fifth-generation
mmWaves	millimeter waves
THz	terahertz
IoT	Internet of Things
BS	base station
SISO	single-input single-output
MISO	multiple-input single-output
MIMO	multiple-input multiple-output
D-IRS	distributed IRS
QoS	quality-of-service
DFT	discrete Fourier transform
SINR	signal-to-noise plus interference ratio
M2M	machine-to-machine communication
LoS	line-of-sight
AODs	angles of departure
TDD	time division duplex
IUABP	IRS-UE association based on pilots
